# Cardiolipin Supports Respiratory Enzymes in Plants in Different Ways

**DOI:** 10.3389/fpls.2017.00072

**Published:** 2017-02-08

**Authors:** Jakob Petereit, Kenta Katayama, Christin Lorenz, Linda Ewert, Peter Schertl, Andreas Kitsche, Hajime Wada, Margrit Frentzen, Hans-Peter Braun, Holger Eubel

**Affiliations:** ^1^Institute of Plant Genetics, Leibniz Universität HannoverHannover, Germany; ^2^Graduate School of Arts and Sciences, The University of TokyoTokyo, Japan; ^3^Graduate School of Agricultural and Life Sciences, The University of TokyoTokyo, Japan; ^4^Leibniz-Institut für Analytische Wissenschaften – ISAS – e.V.Dortmund, Germany; ^5^Institute of Biostatistics, Leibniz Universität HannoverHannover, Germany; ^6^Institute of Biology I, RWTH Aachen UniversityAachen, Germany

**Keywords:** plant mitochondria, cardiolipin, protein complexes, protein supercomplexes, respiration, cytochrome c

## Abstract

In eukaryotes the presence of the dimeric phospholipid cardiolipin (CL) is limited to the mitochondrial membranes. It resides predominantly in the inner membrane where it interacts with components of the mitochondrial electron transfer chain. CL deficiency has previously been shown to affect abundances of the plant NADH-dehydrogenase complex and its association with dimeric cyctochrome c reductase. Using an *Arabidopsis thaliana* knock-out mutant for the final enzyme of CL biosynthesis we here extend current knowledge on the dependence of plant respiration on CL. By correlating respiratory enzyme abundances with enzymatic capacities in mitochondria isolated from wild type, CL deficient and CL complemented heterotrophic cell culture lines a new picture of the participation of CL in plant respiration is emerging. Data indicate a loss of a general reduction of respiratory capacity in CL deficient mitochondria which cannot solely be attributed to decreased abundances or capacities of mitochondrial electron transfer protein complexes and supercomplexes. Instead, it most likely is the result of a loss of the mobile electron carrier cytochrome c. Furthermore, enzymes of the tricarboxylic acid cycle are found to have lower maximum activities in the mutant, including the succinate dehydrogenase complex. Interestingly, abundance of the latter is not altered, indicative of a direct impact of CL deficiency on the enzymatic capacity of this electron transfer chain protein complex.

## Introduction

The dimeric phospholipid cardiolipin (CL) is exclusively found in mitochondria and bacteria. It is of highest concentration in the inner mitochondrial membrane and contributes approximately 10% toward the total lipid content of this organelle (Ardail et al., 1990; Osman et al., 2011). A single CL molecule comprises four acyl chains connected by a bridge of three glycerol moieties, themselves interlinked by two phosphate groups. This composition holds responsible for its wedge-shaped structure in which a bulky fatty acid part is joined to a relatively small polar headgroup. CL synthesis depends on a set of enzymes also involved in the production of other phospholipid species but employs a unique enzyme for the final step of the pathway, cardiolipin synthase (CLS). This enzyme catalyzes the condensation of phosphatidylglycerol (PG) and cytidinediphosphate-diacylglycerol (CDP-DAG) in the mitochondria of animals and plants (Katayama et al., 2004; Chen et al., 2006). Fatty acid composition in CL molecules is variable and up to 23 different CL species have recently been identified across five different plant species (Zhou et al., 2016). Insertion of CL into membrane bilayers induces tensions and membrane bends (Gonzalvez and Gottlieb, 2007). Despite structural differences phosphatidylethanolamine (PE) and CL have overlapping functions, particularly in mitochondrial fusion (Joshi et al., 2012). Due to steric limitations, the CL phosphate groups are more accessible promoting the interactions of CL with proteins (Lewis and McElhaney, 2009).

The mobile electron carrier cytochrome c is an important interaction partner of CL. Studies employing yeast and horse heart cytochrome c revealed alterations in protein conformation upon binding to CL-containing lipid vesicles. The resulting increase in cardiolipin peroxidation capacity is accompanied by higher levels of membrane pore formation and, consequently, cytochrome c leakage (Hanske et al., 2012; Bergstrom et al., 2013). As such, CL: cytochrome c interactions play an important role in the early signaling events ultimately leading to the onset of programmed cell death (PCD) in mammals and yeast (Bergstrom et al., 2013). Recently, Pineau et al. (2013) and Pan et al. (2014) provided strong evidence that CL plays a vital role in protecting plants against PCD induced stresses.

Besides its role as membrane lipid CL also is a structural component of protein complexes and supercomplexes of the inner mitochondrial membrane such as the ADP/ATP carrier (AAC, Nury et al., 2005), the bacterial succinate dehydrogenase complex (II1, Yankovskaya et al., 2003), as well as the eukaryotic bc_1_ complex dimer (III2, Palsdottir et al., 2003), cytochrome c oxidase (IV1, Shinzawa-Itoh et al., 2007) and the ATP synthase complex (V1, Eble et al., 1990). Isolated mitochondrial NADH dehydrogenase complex (I_1_) and III_2_, rely on CL for restoring the respective enzymatic activities after phospholipid depletion (Fry and Green, 1981). In a similar fashion, CL positively influences stability and activity of yeast II_1_ while simultaneously reducing superoxide anion production of II_1_ upon reconstitution in CL-containing nanodiscs (Schwall et al., 2012). In addition, CL is involved in stabilizing yeast respiratory supercomplexes III_2_IV_1_ and III_2_IV_2_, (Zhang et al., 2002; Pfeiffer et al., 2003). Their reconstitution in lipid vesicles also directly depends on CL (Bazan et al., 2013) and other lipids are not or only poorly able to substitute for CL.

Since the bulk of CL research has been conducted on yeast mitochondria and bacteria a picture of the dependence of plant mitochondrial functions on CL is only beginning to emerge. In *Arabidopsis* CL deficiency has recently been linked with (i) disturbances of plant mitochondrial fusion: fission balance, (ii) altered mitochondrial metabolism and (iii) stress tolerance in *Arabidopsis* (Katayama and Wada, 2012; Pineau et al., 2013; Pan et al., 2014). In order to extend current knowledge on the involvement of CL in plant respiration a homozygous knock-out mutant of the final enzyme of CL biosynthesis, CLS, was used for an assessment of respiratory chain components in respect to both, abundance and enzymatic capacity. Data confirm previous results by revealing a strong decrease in supercomplex abundance. In addition, enzyme capacities of the respiratory chain as well as its individual units were quantified and correlated with protein abundances. Results indicate a low reduction in individual enzyme capacities which are mostly due to reduced protein abundances. An exception to this is the succinate dehydrogenase complex, which does not display an altered abundance despite clear reductions in its enzyme capacity. The NADH-producing enzymes of the citric acid cycle are also severely impaired in respect to their enzymatic capacities.

## Results

### Quantitation of CLS in *Arabidopsis* Cell Suspension Cultures of the Homozygous KO-Mutant Line and Its Corresponding Wild Type Line

Stem tissue of the CLS mutant carrying a T-DNA in the CLS gene and a complementing gene copy under an estradiol inducible promotor (Katayama and Wada, 2012) was used to establish cell cultures providing suitable amounts of plant material for organelle preparations. The site of T-DNA insertion is at the start of the fifth exon of the CLS gene (SALK_4984, At4g04870, Katayama and Wada, 2012; Pan et al., 2014). After 7 days of growth cell mass of the homozygous knock-out mutant approximately doubled while the WT cell line increased by factor three (data not shown). Complementation of the *cls* knock-out was induced by adding estradiol to fresh MS-medium to a final concentration of 4 μM prior to subculturing. Organelle preparations were performed after 6 or 7 days of growth.

**FIGURE 1:**
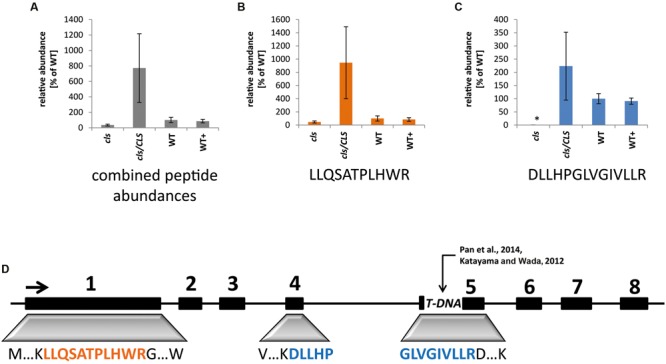
**Quantitation of cardiolipin synthase by means of tSIM-MS.** **(A)** Combined average peak areas for two peptides of At4g04870 (LLQSATPLHWR, DLLHPGLVGIVLLR) in arbitrary units (WT = 100%) for *cls*, *cls/CLS*, WT, and WT+. **(B)** Average peak areas for LLQSATPLHWR. **(C)** Average peak areas for DLLHPGLVGIVLLR. Error bars indicate standard error of three biological replicates, asterisk indicates *p* < 0.05 when compared to WT. **(D)** Position of analyzed peptides within the exon/intron structure of the CLS gene. Boxes indicate exons, lines indicate non-coding regions. TDNA indicates TDNA insertion according to Katayama and Wada (2012) and Pan et al. (2014).

To monitor the success of the T-DNA insertion as well as the complementation strategy in the cell culture system, targeted selected ion monitoring mass spectrometry (tSIM-MS) was performed for two selected tryptic peptides of CLS in isolated mitochondria of the knock-out mutant (*cls*), complemented mutant (*cls/CLS*), wild type (WT), and estradiol treated WT cell cultures (WT+). The peptides (LLQSATPLHWR and DLLHPGLVGIVLLR) were chosen for quantitation due to their traceability in shotgun MS/MS runs, their uniqueness within the *Arabidopsis* proteome, and the low amount of expected modifications due to the absence of cysteines and methionines. The mono-isotopic mass and the following two masses of the isotope distribution of triply charged peptides were taken for this analysis (**Figure 1**). Results indicate comparable amounts of CLS in WT and WT+ but higher average values for *cls/CLS*, concordant with a constitutive expression of the CLS gene in the presence of estradiol. The high level of variation observed for *cls/CLS* is striking and most likely the result of experimental deviations, such as age of estradiol solution. Combined abundances of both peptides reveal CLS abundance in *cls* not changing significantly. However, closer inspection reveals the peptide DLLHPGLVGIVLLR to be virtually absent in *cls*. This peptide is encoded by the end of exon 4 and the start of exon 5 (**Figure 1**, Katayama and Wada, 2012), spanning the insertion site proposed by Pan et al. (2014). The relatively high amounts of LLQSATPLHWR in *cls* in combination with the absence of DLLHPGLVGIVLLR suggest the synthesis of a truncated and therefore inactive form of the CLS protein in *cls.*

### Cardiolipin-Deficiency Disturbs Mitochondrial Matrix Metabolism

**FIGURE 2:**
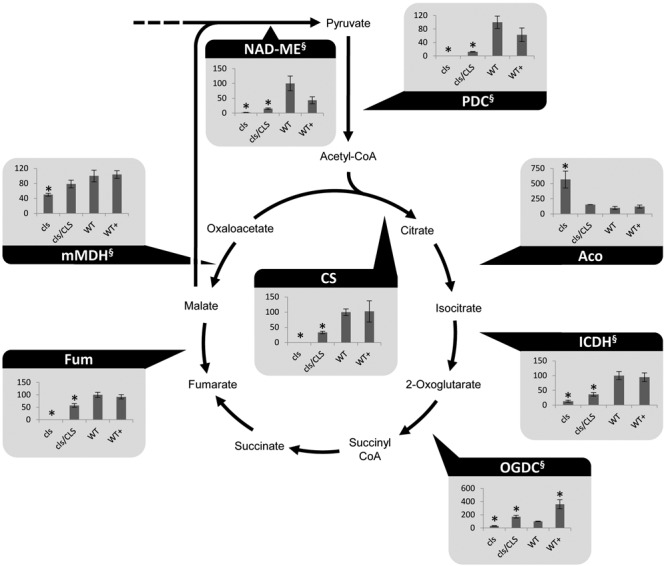
**Photometrically assayed enzymatic capacities of TCA cycle enzymes of three biological replicates of mutant and WT lines.** Arrows indicate the directions of the tested enzymatic reactions. Asterisks indicate *p* < 0.05 compared to WT. Capacities are given as percentages of the corresponding WT values. Of each line three biological replicates were tested, error bars indicate standard deviation. Aco, aconitase; Fum, fumarase; ICDH, isocitrate dehydrogenase; mMDH, mitochondrial malate dehydrogenase; NAD-ME, NAD-malic enzyme; OGDC, 2-oxoglutarate dehydrogenase; PDC, pyruvate dehydrogenase complex; SDH, succinate dehydrogenase complex. ^§^ marks NADH-producing reactions.

Cardiolipin deficiency was reported to have a strong impact on the respiratory capacity of yeast mitochondria but mitochondrial matrix metabolism may be affected as well via feed-back effects. Indeed, altered amounts of TCA-cycle intermediates under CL-deficiency were reported previously (Pineau et al., 2013). Photometric assays for seven TCA-cycle enzymes as well as the pyruvate dehydrogenase complex (PDC) and mitochondrial NAD malic enzyme (NAD-ME) were conducted. Most TCA-cycle enzymes show decreased (2-oxglutarate dehydrogenase complex, OGDC; mitochondrial malate dehydrogenase, mMDH) or severely decreased (PDC; citrate synthase, CS; isocitrate dehydrogenase, ICDH; fumarase, Fum, NAD-ME) capacities in *cls* (**Figure 2**). Hence, all NADH-forming enzymes possess lower capacities in the mutant. Interestingly, aconitase is the only TCA cycle enzyme with increased capacity in the mutant. This enzyme has been shown to be a major regulator of the TCA cycle and its increased capacity may therefore be aimed at increasing flux through this essential pathway (Araujo et al., 2012). Equally interesting is the response of OGDC to estradiol. In both, *cls/CLS* and WT+, capacities of these enzymes are increased considerably compared to the corresponding untreated lines. For mammals, estrogen has been reported to indirectly alter the phosphorylation pattern of OGDC, thereby impacting its function (Lagranha et al., 2010). If this also holds true for OGDC of plant mitochondria is currently unknown but the data presented here seem to support the existence of a similar mechanism. In summary, most TCA cycle components and enzymes associated with this pathway show reduced enzymatic capacities in the mutant. As such, mitochondrial capacity for the production of NADH is reduced under CL deficiency.

### Cardiolipin-Deficiency Lowers Respiratory Capacity of Plant Mitochondria

**FIGURE 3:**
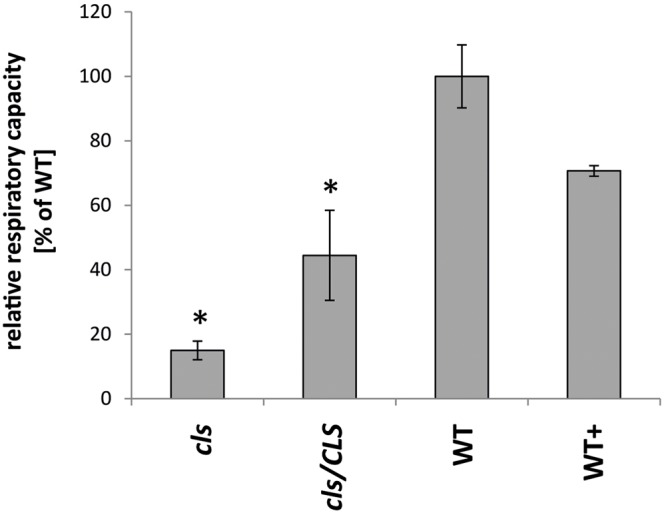
**Respiratory capacities of *cls*, *cls/CLS*, WT, and WT+.** Oxygen consumption rates of lysed mitochondria equaling 1mg of protein were monitored in a Clark-type oxygen electrode in the presence of 1mM NADH. Rates are expressed as arbitrary units of the mean WT rate (100%), error bars indicate standard error of mean of four biological replicates, asterisk indicates *p* < 0.05 when compared to WT.

Analyses of the respiratory capacities of isolated mitochondria by oxygen electrode assays are preferentially conducted using substrates aimed at the internal production of NADH in intact organelles. However, lowered NADH-production within the mutant is expected to interfere with O_2_-consumption rates in the mutant when using such substrates. Hence, the alternative approach of feeding gently disrupted mitochondria with externally applied NADH was chosen to ensure investigation of the true enzymatic capacities of the mitochondrial electron transfer chain (mETC) in *cls, cls/CLS*, and WT. Using osmotic disruption, gently lysed organelles isolated from cell cultures of *cls*, *cls/CLS*, WT, and WT+ were investigated by means of a Clark type oxygen electrode (**Figure 3**). Using the electron donor NADH the electron transfer capacity of *cls* was found to be less than 20% of its WT counterpart (5.6 nmol × min^-1^ × mg_Prot_^-1^/37.1 nmol × min^-1^ × mg_Prot_^-1^). In contrast, *cls/CLS* showed values intermediate to those of *cls* and the WT (16.5 nmol × min^-1^ × mg_Prot_^-1^), indicating a partial recovery of respiratory capacity following the estradiol-triggered complementation of the CLS knock-out. However, given the high abundance of CLS in this line (**Figure 1**) respiratory capacity is unexpectedly low; suggesting that estradiol itself may negatively impact respiratory capacity. Treatment of the WT cell line with this hormone (WT+) also leads to reduced respiratory capacities (26.2 nmol × min^-1^ × mg_Prot_^-1^) compared to untreated WT, thereby supporting the notion of estradiol inhibiting respiration. Indeed, estradiol was previously reported to have detrimental effects on mitochondrial transporters and respiratory chain components in rat liver mitoplasts (Thiede et al., 2012). Hence, respiration rates determined for the estradiol treated lines most likely underestimate their true enzymatic capacities.

### Losses in Enzymatic Capacities of Respiratory Protein Complexes Mostly Match Altered Complex Abundances

**FIGURE 4:**
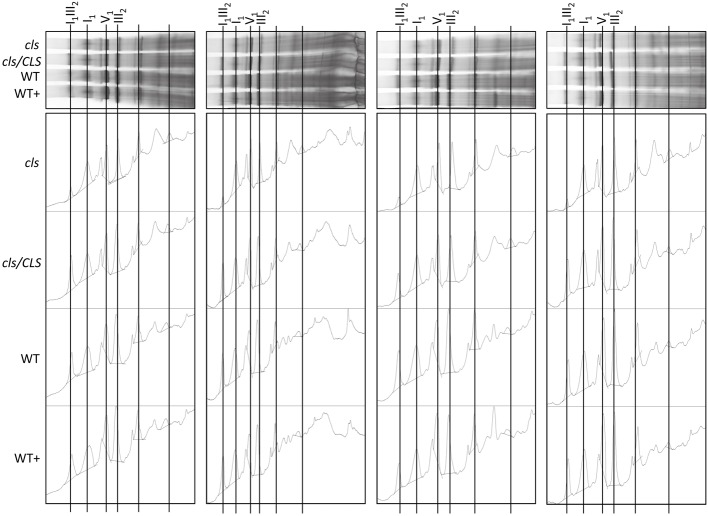
**Abundances of respiratory complexes and supercomplex of four biological replicates in digitonin solubilized mitochondria of *cls, cls/CLS*, WT, and WT+ lines.** Complexes were separated on 1D BN gels **(top)** and protein abundances subsequently assessed using the gel analysis option in the imageJ software **(lower).**

**FIGURE 5:**
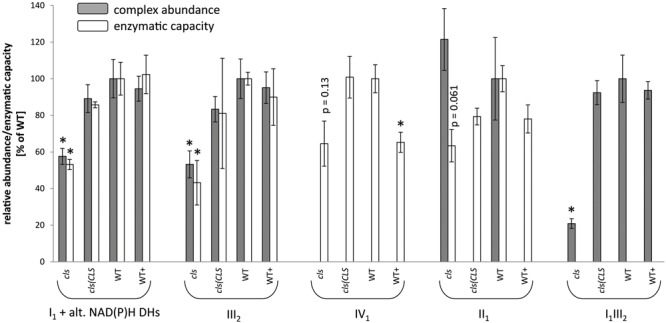
**Relative abundances (gray bars) and enzymatic capacities (white bars) of respiratory complexes I_1_ [including alternative NAD(P)H dehydrogenases], II_1_, III_2_, IV_1_, V_1_ and the I_1_III_2_ supercomplex.** For enzymatic capacities, electron transfer rates from NADH to ferricyanide (I_1_ and potentially I_1_III_2_), succinate to DCIP (II_1_), ubiquinol to oxidized cytochrome C (III_2_ and potentially I_1_III_2_), and reduced cytochrome c to O_2_ (IV_1_) were analyzed photometrically. Abundances of protein complexes and the supercomplex were deduced densitometrically from 1D BN gels (I_1_, III_2_, V_1_ and I_1_III_2_) or from 2D BN/SDS gels (II_1_). All values are shown as arbitrary units of the mean of the respective WT rates. Error bars for enzymatic capacities indicate standard error of mean of three biological replicates consisting of three technical replicates each. Error bars for protein complex abundance indicate standard error of four biological replicates (see **Figure 4**). Asterisks indicate *p* < 0.05 when compared to WT.

**FIGURE 6:**
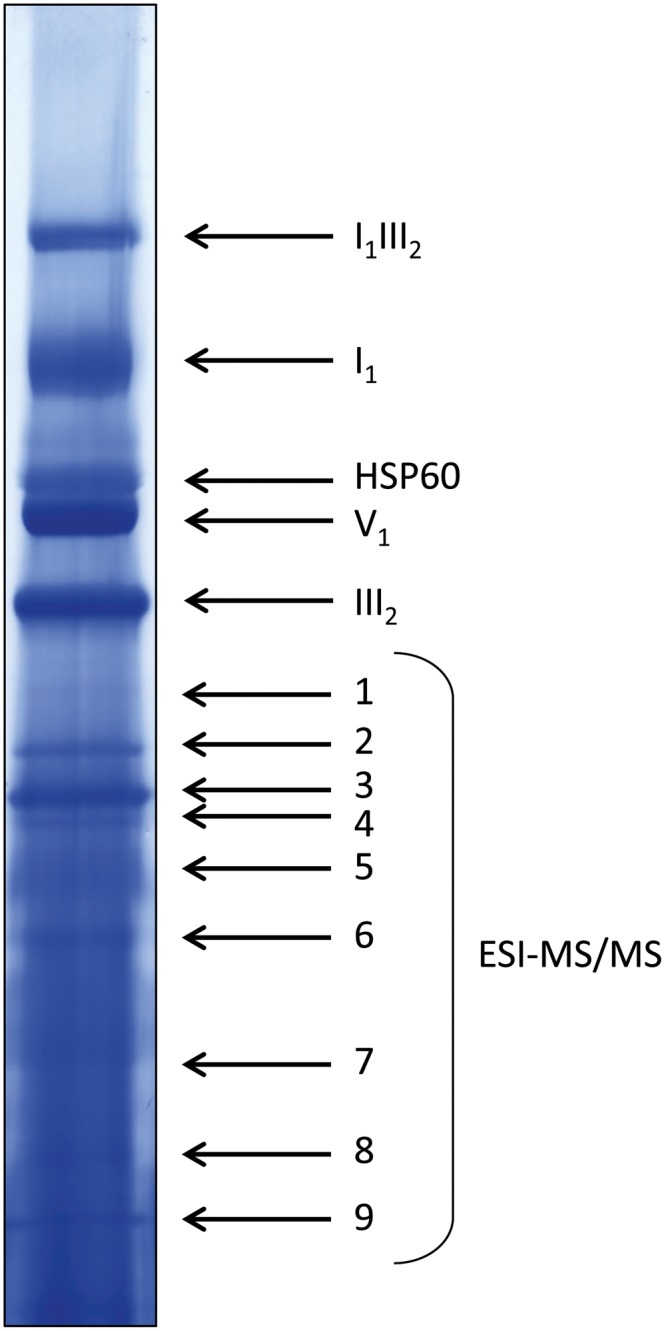
**Coomassie-stained WT lane of a representative 1D BN gel for MS analysis.** Four hundred micrograms of protein were loaded. Bands in the upper half of the lane were identified by comparison to annotated gel lanes, band 1–9 were analyzed by ESI-MS/MS (see Supplementary Table 1).

**FIGURE 7:**
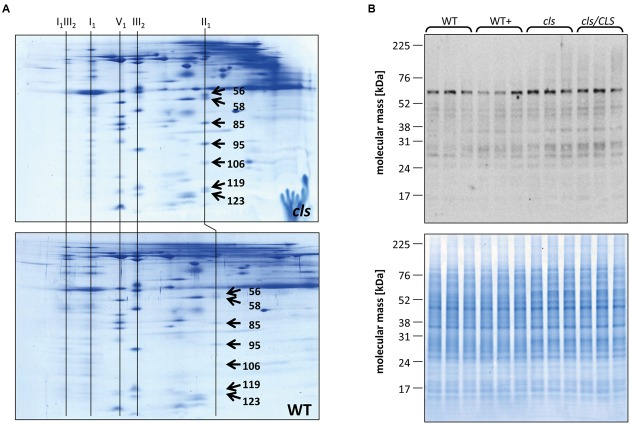
**Quantitation of II_1_ subunits.** **(A)** Representative Coomassie stained BN/SDS gels of cls and WT mitochondria. Spots on gel images were matched and spots volumes were calculated using a commercially available gel analysis tool. The numbers on the spots represent the spot IDs given to the spots by the software. Identities of protein complexes are indicated on top of the gels. **(B)** Immunoblot against the flavoprotein subunit (SDH1-1, AT5g66760, 69.7 KDa) of II_1_ in mutant and WT lines grown in the presence/abscence of estradiol (upper panel). A Coomassie stained gel of cell extracts of the four lines is shown as loading control (lower panel).

Densitometric quantitation of digitonin solubilized I_1,_ III_2_, and the supercomplex consisting of these two individual protein complexes (I_1_III_2_, **Figures 4** and **5**) on 1D BN gels of digitonin treated *cls*, *cls/CLS*, WT, and WT+ mitochondria were performed. In *cls* this supercomplex is decreased to approximately 20% of the WT level while abundances of individual complexes I_1_ and III_2_ are less affected (**Figure 5**). Unfortunately, II_1_ and IV_1_ could not be quantified accurately from 1D BN gels as mass spectrometry (MS) revealed strong background of other proteins in the regions of their respective bands on the gel (**Figure 6**, Supplementary Table 1). To allow quantitation of II_1_ without the risk of including non-II_1_ proteins, spot volumes of the majority of its subunits were obtained from 2D BN/sodium dodecyl sulfate (SDS) gels of WT and *cls* using the Delta2D gel analysis software (Decodon, **Figure 7A**, Supplementary Table 2). Background subtracted volumes of those II_1_ spots detected in six gels (3x WT, 3x *cls*) were then summed up for an estimation of complex abundance and used for the calculation of relative complex abundance. Interestingly, II_1_ does not follow the trend observed for I_1_ and III_2_. On average, abundances of its subunits detectable on BN/SDS gels are higher in *cls* and no subunit of this complex was detected showing a significantly reduced abundance in *cls.* In combination, this may seem indicative of a higher abundance of the assembled complex but statistical analysis does not support this notion since multiplicity adjusted *p*-values equals one suggesting an unchanged abundance (**Table 1**). To further analyze II_1_ abundance in the four lines immunoblots employing an antibody directed against the flavoprotein subunit of II_1_ (SDH1-1, AT5G66760, Peters et al., 2012) were produced using cell extracts of *cls*, *cls/CLS*, WT, and WT+ cell cultures (**Figure 7B**). As can be seen on the Coomassie stained control gel SDH1-1 signals are higher in the *cls* and *cls/CLS* lines but this may be due to higher protein loads of these lines. In summary, the abundance of this complex is best described as being unchanged in *cls* and *cls/CLS*. It follows that the observed reduced enzymatic capacity of this complex in *cls* cannot be attributed to a decreased abundance. Using BN/SDS analyses for I_1_, III_2_, and I_1_III_2_ the changes in protein amount largely match the values obtained from 1D BN gels (**Table 1**). However, multiplicity adjusted *p*-values for I_1_ and III_2_ are notably higher than 0.05. Interestingly, the proportions of the proteins showing a reduction in *cls* for I_1_ and III_2_ are 0.55 and 0.62, respectively. This can most likely be attributed to a compromised spot detection and spot matching by the analysis software which was originally designed for the true round protein spots found on 2D isoelectric focusing (IEF)/SDS gels. Gel spots on BN/SDS differ considerably in shape since they have a rather ellipsoid shape and this compromises spot detection. Particularly I_1_, which is characterized by a rather diffuse band in 1D BN gels and has matching oval spots for its subunits on BN/SDS gels is affected by this. Hence, multiplicity adjusted *p*-values are worse for this complex. No quantitation could be obtained for IV_1_ since this enzyme complex migrates in more than one band on BN/SDS gels and partly overlaps with the TOM-complex (Millar et al., 2004). Its subunits are therefore not confidently detected on 2D BN/SDS gels under the conditions applied here, preventing a meaningful comparative analysis. With the exception of II_1_, data obtained from 1D BN gels are therefore used preferentially over those originating from 2D BN/SDS gels.

**Table 1:** Abundances of respiratory chain protein complexes as derived from 2D BN/SDS PAGE.

	av. ratio WT:*cls*	% of WT	No. of proteins	PropDown	Adjusted *p*-values
**I_1_III_2_**	3.14	32	23	0.9565	0.0146
**I_1_**	1.26	79	21	0.6190	0.3380
**III_2_**	1.16	86	9	0.5556	0.1360
**II_1_**	0.64	156	6	0.0000	1.0000

*av. ratio WT:*cls*, average ratio of subunits within a single protein complex over three biological replicates; % of WT, percentage of average *cls* protein complex abundance as deduced from av. ratio WT:*cls*; No. Of proteins, number of subunits within each complex used for the calculations; PropDown, proportion of subunits of a complex which is significantly reduced; adjusted p-values, multiplicity adjusted p-values for all the subunits of a complex*.

**FIGURE 8:**
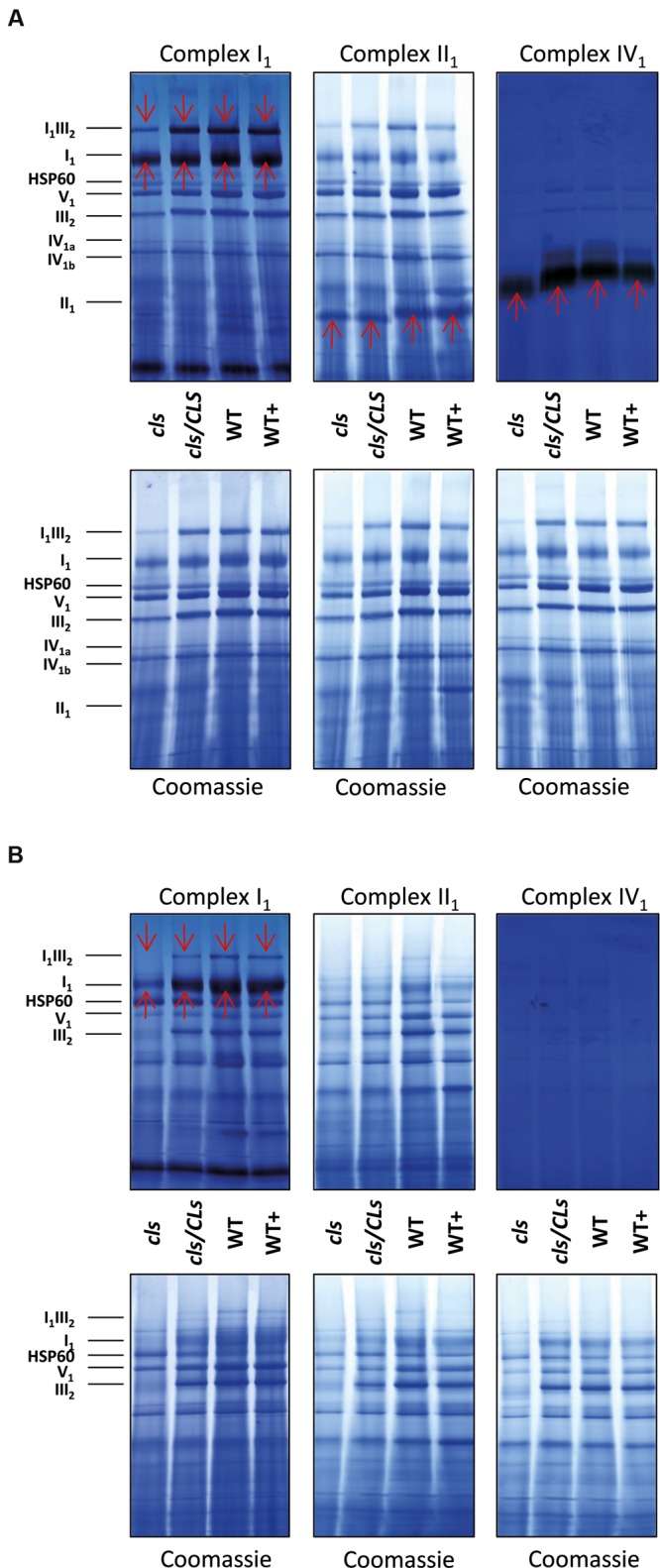
**1D BN gels of mitochondria used for in gel activity assays for complexes I, II, and IV and stained with Coomassie afterward (upper panels) and control Coomassie stained gels (lower panels).** **(A)** Digitonin solubilized mitochondria; **(B)** DDM-solubilized mitochondria. Identities of protein complexes/supercomplexes are given on the right. Red arrows indicate activity stained bands.

In order to gain information on the enzymatic capacities of respiratory complexes *in gel* activity assays for digitonin solubilized I_1_, II_1_, and IV_1_ separated on 1D BN gels were performed (**Figure 8A**). All three complexes display reduced staining intensities in *cls*. Additionally, II_1_ and IV_1_ are characterized by higher electrophoretic mobilities in *cls*. This hints toward lower molecular masses of these complexes. Solubilization of mitochondrial membrane protein complexes with DDM, a stronger detergent than digitonin, reduces mutant protein complex abundances to a higher degree than in the digitonin treated samples (**Figure 8B**). *In gel* activity assays of DDM solubilized protein complexes only worked satisfactorily for I_1_ and residual I_1_III_2_. Here too, reductions are more apparent than in the digitonin solubilized samples. CL deficiency in combination with harsher solubilization therefore suggest a reduced stability of respiratory complexes in the absence of CL.

For a more quantitative assessment of the effect of CL-deficiency on respiratory enzymes their capacities in *cls*, *cls/CLS*, WT, and WT+ were deduced from photometric assays (Birch-Machin et al., 1994; Zhou et al., 2003, **Figure 5**). In *cls*, all four assays revealed reduced enzymatic capacities by 40–60% in comparison to the WT. For complexes I_1_ and III_2_ reductions closely match the changes in protein complex abundances when intensities of the supercomplex bands (I_1_III_2_) are split according to the relation of molecular masses of I_1_ and III_2_ (1000 kD: 500 kD = ^2^/_3_: ^1^/_3_) and added to the abundances of the individual complexes I_1_ and III_2_ (**Figure 5**). Data on IV_1_ abundance are not available and no comment can be made on the correlation of enzyme capacity and protein complex abundance. Interestingly, II_1_ activity is also reduced considerably in *cls* despite the unaltered abundance of this enzyme.

Considering that the transfer of electrons along the mitochondrial respiratory chain is a sequential process and that each of the individual enzymes (protein complexes, supercomplexes) may act as bottlenecks reductions in enzymatic activity of individual complexes do not sufficiently explain the mutant’s strong reduction in respiratory capacity. The electron flow in the mutant’s respiratory chain must therefore face additional restrictions. The I_1_III_2_ supercomplex in the mutant accounts to only 20% of that of the WT (**Figure 5**) and this reduction could have an effect on respiratory capacity.

### Cytochrome c Content of *cls* Mitochondria Is Strongly Reduced

**FIGURE 9:**
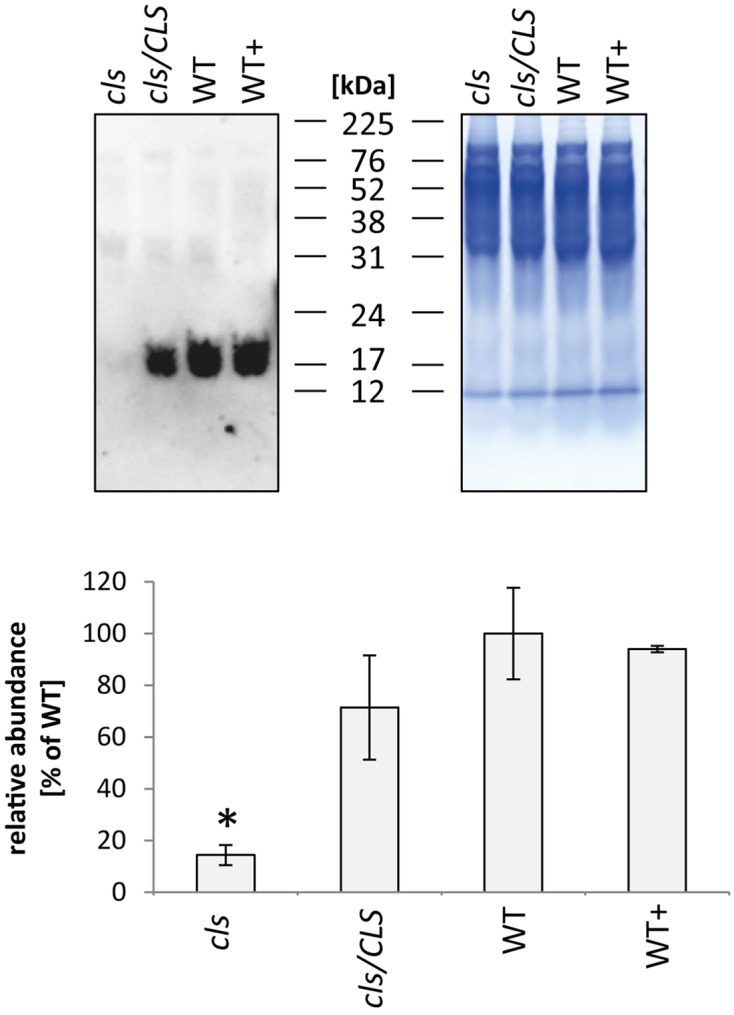
**Quantitation of cytochrome c in mitochondria isolated from *cls*, *cls/CLS*, WT, and WT+ lines by immunoblotting **(top left)**.** Eight micrograms of protein were used of each sample, protein loading was checked by Coomassie stained parallel gel **(top right)**. Quantitation of immune signal **(bottom)** was achieved by densitometric analyses. Cytochrome c abundances are expressed as arbitrary units of the mean of WT (100%). Error bars show standard error of mean for three biological replicates, asterisk indicates *p* < 0.05 when compared to WT.

In addition to the membrane embedded protein complexes of the respiratory chain, the abundance of the mobile electron carrier cytochrome c was analyzed by immunoblotting (**Figure 9**, Welchen et al., 2012). Cytochrome c is reduced to approximately 15% of WT level in *cls* which closely matches the reduction in respiratory capacity. The cytochrome c contents of *cls/CLS* and WT+ are close to WT level, which is not exactly mirrored by their respective respiration rates. However, as mentioned earlier, respiration of *cls/Cls* and WT+ is potentially negatively affected by the estradiol added to the cell culture medium.

## Discussion

The use of a conditional knock-out mutant for the final enzyme of the CL synthesis pathway, CLS, enabled us to further elucidate the dependence of the plant respiratory chain on CL. The data obtained in the course of this study confirm and extend the importance of CL for the processes involved in mitochondrial ATP production in the model plant *Arabidopsis thaliana* by indicating a function of this lipid for protein complex abundance, structure, and enzymatic capacity. In most cases, these alterations are specific for the individual components of the respiratory chain and contribute differently toward the reduced respiratory capacity induced by CL deficiency.

### Implications of CL-Deficiency on ETC Complexes and Supercomplexes

For complexes I_1_ and III_2_, reductions of enzymatic capacities closely follow decreased abundances of these complexes on BN-gels in the mutant, indicative of unchanged enzymatic properties of these complexes under CL deficiency. Most likely, CL can be replaced by other lipids in these complexes under the applied growth conditions without negatively affecting enzymatic capacities. Considering the use of NADH in the I_1_ assay, it should be noted that this also serves as substrate for non-energy conserving alternative NAD(P)H dehydrogenases and that the capacity for NADH oxidation deduced from this assay therefore is not exclusively attributable to singular or supercomplex bound complex I. However, since the assay buffer did not contain calcium which stimulates activity of most NAD(P)H dehydrogenases (Rasmusson et al., 2004) the contribution of alternative dehydrogenases toward the assay results is judged to be negligible. The most abundant plant mitochondrial supercomplex, I_1_III_2_ is of decreased abundance in *cls* and this result is in concordance with previously published data (Pineau et al., 2013). Interestingly, the loss of I_1_III_2_ extends far beyond the reductions observed for the complexes I_1_ and III_2_. In baker’s yeast (which lacks I_1_) abundances of respiratory supercomplexes III_2_IV_1_ and, particularly, III_2_IV_2_ were reduced in a CLS knock-out mutant (Zhang et al., 2002) emphasizing the importance of CL for the abundance of respiratory supercomplexes. While complex IV containing supercomplexes were reported for potato and spinach mitochondria, they are unknown for *Arabidopsis* (Eubel et al., 2004; Krause et al., 2004; Chaban et al., 2014). As such, a direct transfer of results from yeast to *Arabidopsis* is difficult. In combination with the data shown here it nevertheless suggests that CL is more important for the association of respiratory complexes than for the abundance of their building blocks, the individual protein complexes.

It is currently not clear if the lowered amounts of I_1_III_2_ supercomplex in *cls* negatively impact mitochondrial respiration. A reduced level of substrate channeling in the mutant would be the most obvious explanation. However, given that substrate channeling requires a separate, supercomplex bound ubiquinone pool whose existence remains doubtful in the light of recent findings (summarized by Acin-Perez and Enriquez, 2014), it seems unlikely that the association of I_1_ and III_2_ is accountable for the mutant’s lowered respiratory capacity. Hence, other alterations in the CL deficient mETC are expected to be responsible for this, particularly the reduction of mitochondrial cytochrome c content.

### Cytochrome c: CL Interactions

The loss of cytochrome c in the mutant is expected to be the result of missing cytochrome c: CL interactions tying the protein to the inner membrane (Kagan et al., 2009). Some of the changes observed for mitochondrial protein complexes and supercomplexes in the mutant may actually not be caused primarily by the loss of CL but by secondary effects. Reduced cytochrome c content was reported to affect the abundance of IV_1_ but to have little or no implications for I_1_III_2_, I_1_ or III_2_ (Welchen et al., 2012). Quantities of IV_1_ could not be established confidently in this study but have been described to remain unchanged by CL deficiency (Pineau et al., 2013). However, IV_1_ enzymatic capacity is markedly decreased in *cls*. In contrast to IV_1_, II_1_ was reported to be slightly increased in cytochrome c knock-down plants and average II_1_ amounts are also higher in *cls* but variance is too high to confirm this (**Figure 5**).

Once mitochondrial cytochrome c content is reduced to a degree which negatively affects respiration and, consequently, the transport of protons across the inner membrane, the pH of the crista lumina increases. As a result, the binding strength of cytochrome c to anionic phospholipids via exposed lysine residues of the protein decreases. This stabilizes its tertiary structure and lowers the peroxidative capacity of cytochrome c (Rytömaa et al., 1992; Kagan et al., 2009). In turn, cytochrome c, when released from mitochondria, also serves as an antioxidant. The observed reduction in cytochrome c content in *cls* therefore also affects the mitochondrial capacity for scavenging and reducing the production of reactive oxygen species (ROS; Skulachev, 1998; Kagan et al., 2009). Moreover, cytochrome c also is an electron acceptor in the final step of ascorbate synthesis in mitochondria via the L-galactono-1,4-lacton dehydrogenase (GLDH, Schertl et al., 2012; Schimmeyer et al., 2016). Reduction in cytochrome c levels as observed for the mutant may therefore negatively impact the mutant’s ability to detoxify mitochondrial ROS.

### Complex II Behaves Differently to Other mETC Components

Interestingly, and in contrast to all other respiratory chain components II_1_ abundance does not alter between *cls* and WT while its enzymatic capacity is reduced by approximately the same degree seen in I_1_ and III_2_ (**Figure 5**). This connotes a reduction in the activity of each enzymatic unit. Since the flavoprotein subunit (SDH1) depends on activation by ATP (Huang et al., 2010) whose production is compromized in *cls*, *in vivo* activity of mutant II_1_ may be reduced even further in the mutant. CL was found to stabilize yeast II_1_ by strengthening the interaction of the membrane hetero-dimer with the catalytic heterodimer but stability of II_1_ is not affected in *Arabidopsis*. One possible explanation for this may originate from the different solubilization approaches between the two studies: while DDM was chosen for the yeast study the milder digitonin was chosen for protein solubilization here. As suggested for III_2_ DDM may remove potential CL substituting lipids more efficiently than digitonin, thereby decreasing stability of the complexes to a higher degree. Another reason for the stability of II_1_ in *Arabidopsis* may be the higher number of subunits (eight in *Arabidopsis* compared to four in yeast; Eubel et al., 2003; Millar et al., 2004), which may provide a stronger connection between the membrane anchor and the catalytic subunits.

### Other Effects of CL Deficiency on Mitochondrial Metabolism

Reduced NADH oxidation capacity via the respiratory chain must be compensated by deceleration of its production in the mitochondrial matrix in order to limit ROS-production and prevent damage to the organelle and the cell as a whole. This, in turn, requires some sort of feedback inhibition of matrix enzymes. Since in heterotrophic cell culture mitochondria the TCA cycle is understood to operate largely in the cyclic mode, this would require regulation of PDC, ICDH, OGDC, and mMDH, (Lee et al., 2011). Enzymatic assays for these enzymes conducted during the course of this study strongly support this notion. Together with the respiration data this implies a general reduction of main mitochondrial functions in the mutant but further studies will be required to complete the picture of changes induced in plant mitochondria in response to a lack of CL.

### CL in Leaf and Cell Culture Mitochondria

A previous study using the same CLS knock-out line revealed similar results in respect to I_1_III_2_ abundance (Pineau et al., 2013). However, I_1_ abundance in the mutant increased when leaf tissue was treated with digitonin thus contradicting its reduction in *cls* cell culture mitochondria. Interestingly, leaf and cell culture mitochondria display similar effects on I_1_ abundances upon solubilization with DDM since in both tissues, abundances as well as in gel activities of I_1_ are higher in the WT line. These tissue specific differences imply a lower stability of I_1_ in the cell culture upon digitonin solubilization, potentially caused by differing lipid compositions of the inner mitochondrial membranes resulting in differences of the types and amounts of CL substituting lipids.

## Conclusion and Outlook

The importance of lipids has long been neglected in plant mitochondria research and we are currently only beginning to understand the important role mitochondria-specific CL fulfills in this context. This study provides a broad overview of the roles CL fulfills in the respiratory apparatus of plant mitochondria. Earlier studies report an influence of CL on mitochondrial structure. Enzymatic capacities of individual respiratory complexes and supercomplexes of CL deficient mitochondria generally possess decreased protein abundances. The low impact of the absence of CL on individual enzymatic function is possibly due to substitution of CL by other lipids with similar properties. In stark contrast to this, transfer of electrons along the respiratory chain is reduced considerably in the mutant and is beyond the decrease of individual enzyme capacities. The main reason for this is a dramatic reduction in the amount of the mobile electron carrier cytochrome c imposing a severe bottleneck to electron transfer onto molecular oxygen. Interestingly, the succinate dehydrogenase complex (II_1_) stands out from the other respiratory protein complexes by its conflicting data on complex abundance and enzymatic capacity.

Most data on the dependency of respiration on CL have been produced in non-plant model systems but the plant respiratory chain differs significantly from that of yeast or mammals. For example, plant respiratory complexes have a more complex subunit composition and, upon solubilization with digitonin, their supercomplexes show a lesser participation of IV_1_. Additionally, plant respiration is supported by a range of alternative oxidoreductases. For the coordination of all these enzymes in the context of a mitochondrial metabolism undergoing massive changes in response to photosynthetic activity requires mitochondria to maintain a high degree of respiratory ductility. Lipids play a decisive role in the organization of the respiratory chain and the *cls* mutant may provide a toehold to investigate the plasticity of the plant respiratory chain in response to changing environmental and metabolic parameters.

## Materials and Methods

### Plant Material

Seedlings of *cls-2*/*cls-2* harboring pER8:*CLS* plants (here referred to as *cls*, Katayama and Wada, 2012) grown on Murashige and Skoog medium (Murashige and Skoog, 1962) containing 1% (w/v) sucrose and 3% (w/v) phytagel (Sigma–Aldrich, P8169) under continuous light (30 μmol photons m^-2^ s^-1^) at 23^o^C for 1–2 weeks were used for the production of callus materials. When seedlings were large enough to handle, their stems were cut into small pieces, transferred to callus inducing medium [3.1 g L^-1^ Gamborg’s B-5 basal salt mixture (Gamborg et al., 1968; Sigma–Aldrich, G5893), 2% [w/v] glucose, 0.5 g L^-1^ MES pH 5.7 (KOH), 0.1 g L^-1^ *myo*-inositol, 20 mg L^-1^ thiamine-HCl, 1 mg L^-1^ pyridoxine-HCl, 1 mg L^-1^ nicotinic acid, 0.5 mg L^-1^ 2,4-dichlorophenoxyacetic acid, 0.05 mg L^-1^ kinetin, 1 mg L^-1^ biotin, 0.5% [w/v] phytagel (Sigma-Aldrich, P8169)], and incubated at 23°C to generate callus. Calli were then transferred to liquid medium and maintained as described previously (Van Gestel and Verbelen, 2002). Expression of the transgenic copy of CL synthase gene was induced by addition of 17 β-estradiol to fresh MS medium to a final concentration of 4 μM. Cells for mitochondrial preparations were harvested after 6–7 days of sub-culturing.

### Experimental Procedures

#### Isolation of Mitochondria

Mitochondria were isolated from *cls* and WT cell cultures (with and without estradiol supplementation) as outlined by Werhahn et al. (2001) with minor modifications regarding fresh weight and buffer volumes. In brief, approximately 200 g of cells were filtered through two layers of muslin and suspended in 400 mL of ice-cold disruption buffer consisting of 450 mM sucrose, 1.5 mM EGTA, 0.2% [w/v] bovine serum albumin [BSA], 0.6% [w/v] polyvinylpyrrolidone 40, 10 mM dithiothreitol [DTT], 0.2 mM phenylmethylsulfonyl fluoride [PMSF], and 15 mM MOPS [3-(*N*-morpholino)-propanesulfonic acid]/KOH, pH 7.4. Cells were disrupted by three bouts of 15 s each in a Waring commercial blender, separated by intervals of 1 min to allow sedimentation of larger fragments. Prior to isopycnic centrifugation in discontinuous Percoll gradients (18% [v/v], 23% [v/v], and 40% [v/v] Percoll in 0.3 M sucrose, 10 mM MOPS/KOH, pH 7.2, centrifuged for 45 min at 70,000 *g*) mitochondria were enriched by three-step differential centrifugation (3,000 *g* for 5 min [2x] followed by 17,000 *g* for 10 min to pellet the organelles) and resuspended in washing buffer (300 mM sucrose, 1 mM EGTA, 0.2 mM PMSF, and 10 mM MOPS/KOH, pH 7.2). The mitochondrial band taken from the 28% [v/v]: 40% [v/v] Percoll interphase was washed three times in resuspension buffer (0.4 M mannitol, 1 mM EGTA, 0.2 mM PMSF, and 10 mM Tricine/KOH, pH 7.2). Pelleting of organelles was achieved by centrifugation at 12,000 *g* for 10 min.

#### TCA-Cycle Enzyme Assays

For PDC and OGDC assays were performed according to Taylor et al. (2004). Fumarase, citrate synthase, aconitase, ICDH, and mMDH were assayed according to Huang et al. (2015). Capacity of NAD-ME was assayed using a modified protocol of Hatch et al. (1982) in a buffer containing 70 mM HEPES-NaOH (pH 7.2), 360 μM coenzyme A, 20 mM MgCl2, 5mM DTT, 2.5 mM NAD^+^, 100 μM NADH, 2 U malate dehydrogenase, and 120 μg mitochondrial protein. Final results were obtained from three biological replicates.

#### Gel Electrophoreses and in Gel Enzymatic Assays

1D BN-PAGE and 2D BN/SDS-PAGE, Coomassie staining and in gel activity assays were performed as described elsewhere (Eubel et al., 2003, 2004; Behrens et al., 2013). Digitized black and white images of 1D BN gels were used for relative quantitation of protein complexes from four biological replicates with the ‘gel analysis’ option of the ‘Image J’ software^1^. For quantitation of protein complexes from 2D BN/SDS-PAGE, the Delta 2D (Decodon, Greifswald, Germany) software package was employed as described in Welchen et al. (2012). Three replicate gels of each line were produced used for quantitation and variation in gel loading was removed by normalization of the measured abundance values using quantile normalization (Bolstad et al., 2003) for the control and the treatment group separately. To test for differential abundances of protein complexes between the control and the treatment group the ROAST procedure was applied to the log-transformed normalized values which allows for protein-wise correlations within a protein complex (Wu et al., 2010). In addition to multiplicity adjusted *p*-values (Holm, 1979) the proportion of proteins that contribute to the differential abundances between the control and treatment group was calculated. All calculations were performed using the statistical software R and its add-on package LIMMA.

#### Western Blotting

Eight micrograms of mitochondrial protein were supplemented with Laemmly loading buffer and separated on 10-comb precast 10% [w/v] to 20% [w/v] acrylamide gradient gels (Life Technologies, Darmstadt, Germany). Proteins were transferred to reinforced nitrocellulose membranes (Optitran BA-S 83, GE Healthcare, Freiburg, Germany) using semi-dry blotting (Trans-Blot SD Cell, Biorad, Munich, Germany) for 90 min in transfer buffer [37 mM glycine, 130 mM tris(hydroxymethyl)aminomethane, 0.275% [w/v] SDS, 20% [v/v] methanol]. For cytochrome c quantitation monoclonal antibodies directed against pigeon cytochrome c (7H8.2C12, Pharmingen, San Diego, CA, USA) in a 1:1000 dilution were used to probe the western blots. Signal generation was then achieved using anti-mouse secondary antibodies conjugated with horseradish peroxidase (HRP) via chemiluminescence in a buffer containing 100 mM Tris pH 8.5, 0.23 mM p-coumaric acid, 1.25 mM luminol, 0.00015% [v/v] H_2_O_2_. Quantitation of signals from three biological replicates was performed using the ‘image J’ software. For SDH1-1 quantitation, polyclonal antibodies (Peters et al., 2012) in a 1:1000 dilution were used and subsequently detected via biotin-conjugated goat-anti-rabbit secondary antibodies. Chemiluminescence signals of three biological replicates were generated by incubation with avidin-conjugated HRP via as described above.

#### Respiratory Assays

One hundred micrograms of mitochondrial protein (according to Bradford, 1976) were assayed in a Clark-type oxygen electrode. Transport of electrons from externally applied NADH along the respiratory chain was measured on disrupted organelles. Since repeated freeze/thawing was suspected to have detrimental effects on supercomplex stability mitochondria were taken up in H_2_O to promote osmotic lysis. Oxygen consumption rates of four biological replicates were then recorded in the presence of 1 mM NADH, sequentially supplemented with KCN and *n*-propyl gallate (nPG) to final concentrations of 1 and 0.5 mM, respectively.

#### Respiratory Chain Enzyme Assays

Analyses of enzymatic capacities of respiratory chain protein complexes were carried out as described by Birch-Machin et al. (1994; complexes II, III, and IV) and Zhou et al. (2003; complex I) respectively, using frozen mitochondria and reaction volumes of 300 μl per assay. Protein concentrations of the samples were established using the Bradford assay (Bradford, 1976). Analyses of three biological replicates consisting of three technical replicates each were carried out in an Epoch Microplate Reader (Biotek, Winooski, VT, USA). Readings containing all components (including the mitochondria) except the substrates were performed as negative controls. In short, the experiments were carried out as follows:

Complex I capacities were assayed in reaction buffer containing 50 mM Tris/HCl pH 7.4, 0.5 mM K_3_Fe(CN)_6_, 0.2 mM NADH, supplemented with 2 μg mitochondrial protein. Reduction of K_3_Fe(CN)_6_ was recorded at 420 nm and enzymatic activity was determined using *E* = 1 mM^-1^ × cm^-1^.

For analyzing complex II capacities 15 μg of mitochondrial protein were incubated for 5 min in 50 mM Tris/HCl pH 7.4, 20 mM succinate, 5 mM MgCl_2_, and 0.3 mM ATP, followed by addition of 2 mM KCN, 0.5 mM salicylhydroxamic acid (SHAM) and 100 μM decylubiquinone. After starting the reaction by adding 50 μM dichlorindophenol (DCIP) its reduction was monitored at 600 nm. Enzymatic activity was calculated using *E* = 19.1 mM^-1^ × cm^-1^.

Complex III_2_ capacities were analyzed by following the reduction of cytochrome c in a reaction buffer containing 50 mM Tris/HCl, 5 mM MgCl_2_, 2 mM KCN, 30 μM cytochrome c, and 100 μM reduced decylubiquinone (reduction by sodium borate). Two micrograms of mitochondrial protein were used per assay. The reaction was monitored at 550 nm, enzymatic rates were calculated using *E* = 19 mM^-1^ × cm^-1^.

Capacities of complex IV were assayed by recording oxidation rates of cytochrome c in a buffer containing 50 mM Tris/HCl pH 7.4, 0.3 mM dodecylmaltoside, and 15 μM cytochrome c (reduction by sodium dithionate). One microgram mitochondrial protein was used per assay. The reaction was monitored at 550 nm and enzymatic rates were calculated using *E* = 19 mM^-1^ × cm^-1^.

#### Mass Spectrometry – Sample Preparation and Data-Dependent Acquisition Tandem MS

These procedures were carried out as described in Fromm et al. (2016). In brief, proteins bands were cut using a scalpel and diced to yield cubes of approximately 1.5 mm. Proteins were first reduced by DTT, followed by alkylation of cysteine residues with iodoacetamide, tryptic digestion and peptide extraction. Prior to MS, samples were reconstituted in 20 μl of a solution containing 2% [v/v] ACN, 0.1% [v/v] formic acid (FA).

Tandem mass spectrometry (MS/MS) analysis was performed by means of a Q-Exactive (Thermo Fisher Scientific, Dreieich, Germany) mass spectrometer coupled to a Ultimate 3000 (Thermo Fisher Scientific, Dreieich, Germany) UPLC. Peptides were eluted from the C18 reverse phase column during a 60 min Acetonitrile gradient (2–30%). For UPLC and MS settings please refer to Fromm et al. (2016). MS/MS data were queried against an in-house TAIR10 database, modified to also include common contaminants (keratin, trypsin), MS-standards (BSA, fibrinopeptide) and known modifications of mitochondrial encoded proteins based on RNA-editing (AGIs) using Proteome Discoverer (Thermo Fisher Scientific, Dreieich, Germany). Search runs employed the Mascot software (Matrix Science, London, United Kingdom). Only the best ten hits for each band are shown. Frequent contaminants (BSA, keratin, trypsin) were removed from the final output lists.

#### Mass Spectrometry – tSIM of CLS Peptides

Sample preparation was performed as outlined above with minor changes. Fifty microgram of protein were loaded onto a classical Laemmli glycine/SDS gel (Laemmli, 1970) which ran until the proteins focused at the stacking gel/separating gel border. After Coomassie staining, the protein band was cut out and digested as described above. The MS was run in positive ion mode, MS spectra of selected peptide mass/charge ratios (441.5850 for LLQSATPLHWR, and 505.9864 for DLLHPGLVGIVLLR) were recorded from 15 to 95 min with the resolution set to 70000, AGC target to 1e5, maximum injection time to 400 ms, the scan range to 300 to 1200 m/z, loop count to 1, isolation window to 1, and msx count to 4. For dd-MS2, the resolution was set to 17500, AGC target to 1e5, maximum injection time to 1000 ms, Loop count to 8, isolation window to 1.0 m/z, fixed first mass to 100.0 m/z, and NCE to 27.0 (stepped NCE deactivated). Data dependent (dd) settings were as follows: underfill ratio, 0.1%; intensity threshold, 1.0E2; apex trigger, 10–60 s; charge exclusion, unassigned, 1, 5, 5 – 8, >8; peptide match, preferred; exclude isotopes, off; dynamic exclusion, 40.0 s.

MS/MS data were queried against an in-house TAIR10 database as described above. Quantitation of peptides of three biological replicates was performed using the PinPoint software (Thermo Fisher Scientific, Dreieich, Germany) v1.4.0 using the following conditions: peak width set to 1.00 min, minimum signal threshold to 10000, possible alignment error to 1.0 min, number of smoothing points to 30. WT was chosen as reference group and was arbitrarily declared to amount to 1 fmol. Weighting scheme for quantitation was set to 1/x. Selection of peaks for quantitation was checked and, if necessary, manually adjusted to match MS/MS spectra. Complete peak areas were calculated on smoothed data. Raw data were extracted from PinPoint and submitted to normalization by the number of recorded MS1 scans as extracted from the RawMeat software (VAST Scientific^2^). Normalized peak areas for combined and individual peptides were averaged within each cell culture line and the standard error was calculated.

## Author Contributions

JP, KK, CL, LE, and PS provided the experimental data presented in this manuscript. AK provided support for statistical analyses. HW, MF, H-PB and HE were involved in the planning and supervision of experiments. All members participated in preparation of the manuscript.

## Conflict of Interest Statement

The authors declare that the research was conducted in the absence of any commercial or financial relationships that could be construed as a potential conflict of interest.

## Footnotes

1 http://imagej.nih.gov/ij

1 www.vastscientific.com
