# Supplementary material for: Cardiolipin Supports Respiratory Enzymes in Plants in Different Ways
Source: Front Plant Sci. 2017 Feb 8;8:72. doi: 10.3389/fpls.2017.00072 (PMC5296312; doi:10.3389/fpls.2017.00072)
Supplement: Supplementary file 1 [file Table_1.PDF]

# 1

| Accession | Description | # Peptides | # PSMs | Coverage | Score |
|---|---|---|---|---|---|
| AT4G00570.1 | NAD-dependent malic enzyme 2 | 31 | 69 | 72.49 % | 2530,0 |
| AT3G23990.1 | heat shock protein 60 | 31 | 65 | 64.12 % | 2351,5 |
| ATMG01190.E | RNA Edit ATP1 ATP synthase subunit 1 | 21 | 51 | 55.03 % | 1684,0 |
| AT3G13860.1 | heat shock protein 60-3A | 21 | 40 | 50.87 % | 1609,2 |
| AT2G33210.1 | heat shock protein 60-2 | 26 | 50 | 53.68 % | 1605,9 |
| AT2G13560.1 | NAD-dependent malic enzyme 1 | 20 | 44 | 53.93 % | 1600,1 |
| AT5G08670.1 | ATP synthase alpha/beta family protein | 22 | 47 | 55.22 % | 1459,2 |
| AT4G37910.1 | mitochondrial heat shock protein 70-1 | 19 | 31 | 36.80 % | 1110,0 |
| AT2G47510.1 | fumarase 1 | 10 | 19 | 28.86 % | 709,5 |
| AT3G08580.1 | ADP/ATP carrier 1 | 9 | 18 | 28.08 % | 708,9 |

# 2

| Accession | Description | # Peptides | # PSMs | Coverage | Score |
|---|---|---|---|---|---|
| AT4G00570.1 | NAD-dependent malic enzyme 2 | 40 | 308 | 84.35 % | 10189,6 |
| AT2G13560.1 | NAD-dependent malic enzyme 1 | 31 | 200 | 66.61 % | 5992,9 |
| AT5G08670.1 | ATP synthase alpha/beta family protein | 26 | 135 | 63.49 % | 4213,0 |
| AT3G23990.1 | heat shock protein 60 | 35 | 105 | 70.02 % | 3520,2 |
| ATMG01190.E | RNA Edit ATP1 ATP synthase subunit 1 | 23 | 95 | 56.80 % | 2852,7 |
| AT2G33210.1 | heat shock protein 60-2 | 30 | 85 | 59.15 % | 2640,8 |
| AT3G13860.1 | heat shock protein 60-3A | 24 | 58 | 57.52 % | 2215,8 |
| AT4G37910.1 | mitochondrial heat shock protein 70-1 | 22 | 41 | 40.62 % | 1395,7 |
| AT3G08580.1 | ADP/ATP carrier 1 | 10 | 26 | 28.35 % | 989,4 |
| AT3G55410.1 | 2-oxoglutarate dehydrogenase, E1 component | 23 | 26 | 31.47 % | 835,0 |

# 3

| Accession | Description | # Peptides | # PSMs | Coverage | Score |
|---|---|---|---|---|---|
| AT5G08670.1 | ATP synthase alpha/beta family protein | 34 | 939 | 78.78 % | 32096,8 |
| ATMG01190.E | RNA Edit ATP1 ATP synthase subunit 1 | 29 | 825 | 60.95 % | 28056,7 |
| AT2G07698.1 | ATPase, F1 complex, alpha subunit protein | 25 | 772 | 35.78 % | 26097,9 |
| AT2G33040.1 | gamma subunit of Mt ATP synthase | 20 | 143 | 54.15 % | 4902,9 |
| AT4G37910.1 | mitochondrial heat shock protein 70-1 | 31 | 81 | 52.20 % | 3255,8 |
| AT3G08580.1 | ADP/ATP carrier 1 | 17 | 63 | 39.90 % | 2035,8 |
| AT2G47510.1 | fumarase 1 | 15 | 44 | 51.83 % | 1800,8 |
| ATMG00640.E | RNA Edit ORF25 hydrogen ion transporting ATP synthases | 9 | 58 | 42.19 % | 1752,5 |
| AT3G52300.1 | ATP synthase D chain, mitochondrial | 21 | 61 | 84.52 % | 1634,7 |
| AT3G55410.1 | 2-oxoglutarate dehydrogenase, E1 component | 31 | 45 | 41.59 % | 1419,2 |

# 4

| Accession | Description | # Peptides | # PSMs | Coverage | Score |
|---|---|---|---|---|---|
| AT5G23140.1 | nuclear-encoded CLP protease P7 | 12 | 155 | 53.53 % | 4477,7 |
| AT5G08670.1 | ATP synthase alpha/beta family protein | 26 | 96 | 64.39 % | 3341,9 |
| AT4G37910.1 | mitochondrial heat shock protein 70-1 | 29 | 75 | 53.37 % | 2908,3 |
| ATMG01190.E | RNA Edit ATP1 ATP synthase subunit 1 | 24 | 75 | 56.21 % | 2626,0 |
| ATMG00640.E | RNA Edit ORF25 hydrogen ion transporting ATP synthases | 12 | 74 | 50.52 % | 2288,2 |
| AT2G47510.1 | fumarase 1 | 18 | 51 | 55.08 % | 2093,9 |
| AT3G52300.1 | ATP synthase D chain, mitochondrial | 21 | 68 | 89.29 % | 1857,7 |
| AT3G48000.1 | aldehyde dehydrogenase 2B4 | 22 | 43 | 54.09 % | 1667,3 |
| AT3G08580.1 | ADP/ATP carrier 1 | 17 | 50 | 38.85 % | 1649,6 |
| AT5G26860.1 | lon protease 1 | 32 | 45 | 43.09 % | 1505,7 |

## 5

| Accession | Description | # Peptides | # PSMs | Coverage | Score |
|---|---|---|---|---|---|
| gi162648 | albumin BSA [Bos taurus] | 17821,22 | 54,37 | 30,0 | 649,0 |
| gi1346343 | Keratin, type II cytoskeletal 1 | 3906,03 | 44,41 | 24,0 | 129,0 |
| AT5G18170.1 | glutamate dehydrogenase 1 | 3224,63 | 44,53 | 16,0 | 132,0 |
| AT5G07440.1 | glutamate dehydrogenase 2 | 2617,49 | 43,07 | 15,0 | 103,0 |
| gi547749 | Keratin, type I cytoskeletal 10 | 1962,87 | 41,82 | 21,0 | 49,0 |
| AT3G20000.1 | translocase of the outer mitochondrial membrane 40 | 1929,21 | 62,14 | 13,0 | 85,0 |
| AT3G48000.1 | aldehyde dehydrogenase 2B4 | 1832,74 | 47,03 | 21,0 | 55,0 |
| gi547754 | Keratin, type II cytoskeletal 2 epidermal | 1615,12 | 39,22 | 24,0 | 53,0 |
| AT3G55410.1 | 2-oxoglutarate dehydrogenase, E1 component | 1381,16 | 32,65 | 29,0 | 46,0 |
| AT3G01280.1 | voltage dependent anion channel 1 | 1209,19 | 46,38 | 12,0 | 39,0 |

## 6

| Accession | Description | # Peptides | # PSMs | Coverage | Score |
|---|---|---|---|---|---|
| AT5G07440.1 | glutamate dehydrogenase 2 | 25 | 260 | 73.24 % | 7609,3 |
| AT3G48000.1 | aldehyde dehydrogenase 2B4 | 27 | 188 | 61.90 % | 5587,1 |
| AT5G18170.1 | glutamate dehydrogenase 1 | 21 | 162 | 54.26 % | 4569,8 |
| AT2G47510.1 | fumarase 1 | 21 | 77 | 60.98 % | 2808,2 |
| AT5G26860.1 | lon protease 1 | 41 | 91 | 50.43 % | 2619,5 |
| AT3G55410.1 | 2-oxoglutarate dehydrogenase, E1 component | 42 | 91 | 48.87 % | 2591,2 |
| AT4G37910.1 | mitochondrial heat shock protein 70-1 | 28 | 61 | 50.00 % | 2095,3 |
| AT5G65750.1 | 2-oxoglutarate dehydrogenase, E1 component | 33 | 71 | 43.41 % | 2027,2 |
| AT3G07770.1 | HEAT SHOCK PROTEIN 89.1 | 34 | 60 | 53.32 % | 1827,9 |
| AT5G08670.1 | ATP synthase alpha/beta family protein | 20 | 39 | 55.58 % | 1301,1 |

## 7

| Accession | Description | # Peptides | # PSMs | Coverage | Score |
|---|---|---|---|---|---|
| AT1G79440.1 | aldehyde dehydrogenase 5F1 | 42 | 345 | 68.18 % | 10885,6 |
| AT5G26780.1 | serine hydroxymethyltransferase 2 | 35 | 263 | 74.47 % | 8587,0 |
| AT3G07770.1 | HEAT SHOCK PROTEIN 89.1 | 51 | 181 | 69.71 % | 7175,4 |
| AT3G08580.1 | ADP/ATP carrier 1 | 25 | 286 | 54.07 % | 7121,3 |
| AT5G66760.1 | succinate dehydrogenase 1-1 | 28 | 192 | 61.83 % | 6671,0 |
| AT4G37930.1 | serine transhydroxymethyltransferase 1 | 28 | 145 | 71.95 % | 5119,5 |
| AT5G62530.1 | aldehyde dehydrogenase 12A1 | 33 | 150 | 67.81 % | 4635,8 |
| AT3G55410.1 | 2-oxoglutarate dehydrogenase, E1 component | 51 | 126 | 55.06 % | 4390,9 |
| AT4G37910.1 | mitochondrial heat shock protein 70-1 | 36 | 93 | 58.80 % | 4181,0 |
| AT4G39690.1 | unknown function protein | 42 | 108 | 75.08 % | 4157,2 |

## 8

| Accession | Description | # Peptides | # PSMs | Coverage | Score |
|---|---|---|---|---|---|
| AT1G79440.1 | aldehyde dehydrogenase 5F1 | 42 | 378 | 67.80 % | 12252,4 |
| AT5G26780.1 | serine hydroxymethyltransferase 2 | 30 | 231 | 71.95 % | 7719,1 |
| AT5G66760.1 | succinate dehydrogenase 1-1 | 28 | 189 | 66.40 % | 6689,3 |
| AT3G07770.1 | HEAT SHOCK PROTEIN 89.1 | 48 | 176 | 67.83 % | 6556,6 |
| AT3G29320.1 | Glycosyl transferase, family 35 | 47 | 113 | 66.11 % | 4682,5 |
| AT4G37930.1 | serine transhydroxymethyltransferase 1 | 25 | 126 | 59.38 % | 4487,0 |
| AT1G26460.1 | Tetratricopeptide repeat (TPR)-like superfamily protein | 33 | 124 | 73.02 % | 4303,9 |
| AT4G37910.1 | mitochondrial heat shock protein 70-1 | 34 | 95 | 57.62 % | 3977,3 |
| AT4G34200.1 | D-3-phosphoglycerate dehydrogenase | 24 | 98 | 47.60 % | 3868,2 |
| AT3G55410.1 | 2-oxoglutarate dehydrogenase, E1 component | 47 | 125 | 55.95 % | 3867,8 |

9

| Accession | Description | # Peptides | # PSMs | Coverage | Score |
|---|---|---|---|---|---|
| AT1G79440.1 | aldehyde dehydrogenase 5F1 | 42 | 378 | 67.80 % | 12252,4 |
| AT5G26780.1 | serine hydroxymethyltransferase 2 | 30 | 231 | 71.95 % | 7719,1 |
| AT5G66760.1 | succinate dehydrogenase 1-1 | 28 | 189 | 66.40 % | 6689,3 |
| AT3G07770.1 | HEAT SHOCK PROTEIN 89.1 | 48 | 176 | 67.83 % | 6556,6 |
| AT3G29320.1 | Glycosyl transferase, family 35 | 47 | 113 | 66.11 % | 4682,5 |
| AT4G37930.1 | serine transhydroxymethyltransferase 1 | 25 | 126 | 59.38 % | 4487,0 |
| AT1G26460.1 | Tetratricopeptide repeat (TPR)-like superfamily protein | 33 | 124 | 73.02 % | 4303,9 |
| AT4G37910.1 | mitochondrial heat shock protein 70-1 | 34 | 95 | 57.62 % | 3977,3 |
| AT4G34200.1 | D-3-phosphoglycerate dehydrogenase | 24 | 98 | 47.60 % | 3868,2 |
| AT3G55410.1 | 2-oxoglutarate dehydrogenase, E1 component | 47 | 125 | 55.95 % | 3867,8 |

Suppl. Tab. 1: Tandem MS results of bands 1 to 9 in Suppl. Fig. 3. Only the top ten proteins with the highest scores are shown. Accession, Arabidopsis genome identifier; Description, protein function according to The Arabidopsis Information Resource (TAIR); # Peptides, number of unique peptides; # PSM, number of times peptides of the corresponding protein have been selected for tandem MS; Coverage, portion of the protein sequence covered by the identified peptides in percent; Score, MASCOT score.
