# Supplementary material for: Cardiolipin Supports Respiratory Enzymes in Plants in Different Ways
Source: Front Plant Sci. 2017 Feb 8;8:72. doi: 10.3389/fpls.2017.00072 (PMC5296312; doi:10.3389/fpls.2017.00072)
Supplement: Supplementary file 2 [file Table_2.pdf]

Supplemental Table 2: integrated spot volumes of II_1 subunits

spot	integrated gray volumes w/o background - *cls*			integrated gray volumes w/o background - WT		
	1	2	3	1	2	3
56	389,7	874,3	727,7	783,1	305,7	601,2
58	364,6	1142,0	859,0	607,6	336,0	620,2
83	1045,1	1076,6	1351,8	1261,7	534,6	1259,5
95	781,9	1079,6	1270,5	1073,8	548,2	1077,5
106	577,3	612,9	757,0	nd	292,6	579,3
119	270,3	373,4	212,4	424,7	65,0	330,5
123	497,2	606,0	964,1	720,7	321,5	559,1
sum (w/o spot 106)	3348,8	5151,9	5385,4	4871,6	2111,0	4447,9
